# Higher Plasma Viremia in the Febrile Phase Is Associated With Adverse Dengue Outcomes Irrespective of Infecting Serotype or Host Immune Status: An Analysis of 5642 Vietnamese Cases

**DOI:** 10.1093/cid/ciaa1840

**Published:** 2020-12-19

**Authors:** Nguyen Lam Vuong, Nguyen Than Ha Quyen, Nguyen Thi Hanh Tien, Nguyen Minh Tuan, Duong Thi Hue Kien, Phung Khanh Lam, Dong Thi Hoai Tam, Tran Van Ngoc, Sophie Yacoub, Thomas Jaenisch, Ronald B Geskus, Cameron P Simmons, Bridget A Wills

**Affiliations:** 1 Oxford University Clinical Research Unit, Hospital for Tropical Diseases, Ho Chi Minh City, Vietnam; 2 University of Medicine and Pharmacy at Ho Chi Minh City, Ho Chi Minh City, Vietnam; 3 Children’s Hospital No. 1, Ho Chi Minh City, Vietnam; 4 Hospital for Tropical Diseases, Ho Chi Minh City, Vietnam; 5 Centre for Tropical Medicine and Global Health, Nuffield Department of Clinical Medicine, University of Oxford, Oxford, United Kingdom; 6 Section of Clinical Tropical Medicine, Heidelberg University Hospital, Heidelberg, Germany; 7 Institute for Vector-Borne Disease, Monash University, Clayton, Australia

**Keywords:** dengue, viremia, severe dengue, plasma leakage, hospitalization

## Abstract

**Background:**

One of the generally accepted constructs of dengue pathogenesis is that clinical disease severity is at least partially dependent upon plasma viremia, yet data on plasma viremia in primary versus secondary infections and in relation to clinically relevant endpoints remain limited and contradictory.

**Methods:**

Using a large database comprising detailed clinical and laboratory characterization of Vietnamese participants enrolled in a series of research studies executed over a 15-year period, we explored relationships between plasma viremia measured by reverse transcription–polymerase chain reaction and 3 clinically relevant endpoints—severe dengue, plasma leakage, and hospitalization—in the dengue-confirmed cases. All 4 dengue serotypes and both primary and secondary infections were well represented. In our logistic regression models we allowed for a nonlinear effect of viremia and for associations between viremia and outcome to differ by age, serotype, host immune status, and illness day at study enrollment.

**Results:**

Among 5642 dengue-confirmed cases we identified 259 (4.6%) severe dengue cases, 701 (12.4%) patients with plasma leakage, and 1441 of 4008 (40.0%) patients recruited in outpatient settings who were subsequently hospitalized. From the early febrile phase onwards, higher viremia increased the risk of developing all 3 endpoints, but effect sizes were modest (ORs ranging from 1.12–1.27 per 1-log increase) compared with the effects of a secondary immune response (ORs, 1.67–7.76). The associations were consistent across age, serotype, and immune status groups, and in the various sensitivity and subgroup analyses we undertook.

**Conclusions:**

Higher plasma viremia is associated with increased dengue severity, regardless of serotype or immune status.

## Teaser

From the early febrile phase onwards, higher plasma viremia increases the risk of subsequent progression to adverse dengue outcomes, with little evidence for effect modification by potential confounders. However, the effect sizes are modest compared with a secondary immune response.

In 2019 dengue, the most common mosquito-borne viral pathogen of humans, was identified by the World Health Organization (WHO) as 1 of the top 10 threats to global health [1]. Disease incidence has increased progressively over the last 50 years, with approximately 100 million dengue virus (DENV) infections now estimated to occur annually across more than 100 countries, including approximately 50 million febrile disease cases [2].

Most symptomatic infections result in a self-limiting nonspecific viral syndrome, but a small minority of patients experience more severe manifestations. Of particular concern is a vasculopathy that causes plasma leakage and may progress to potentially fatal dengue shock syndrome (DSS) [3, 4]. All 4 DENV serotypes can cause severe disease, but the risk for complications increases during second or subsequent infections. Following a primary infection, long-lived immunity develops against the infecting serotype, but protection against heterologous serotypes is short lived [4]. During a subsequent heterotypic (secondary) infection, antibody dependent enhancement (ADE), acting in concert with altered T-cell responses, increases the overall viral burden [5–9], resulting in downstream adverse effects on vascular function. Strong evidence supports a role for DENV nonstructural protein 1 (NS1) in disrupting the integrity of the endothelial barrier [10, 11], with a range of cytokines, mast cell products, and lipid mediators also implicated in the pathogenesis of the vasculopathy [12].

Most complications manifest during a critical period approximately 4–6 days from symptom onset, as both fever and viremia are resolving [13], supporting the view that immunological mechanisms play a role. However, data on plasma viremia in primary versus secondary infections and in relation to clinical disease severity remain limited and contradictory. The magnitude and duration of viremia are variously reported as being greater in primary than in secondary infections [14–17], in secondary than in primary infections [18], or to be comparable in both groups [19]. Similarly, some studies suggest that higher viremia is associated with severe outcomes [14–16, 20, 21], while others report no relationship between viremia and severity [18, 19, 22–25]. However, many of these studies were small and did not account for the rapidly changing viremia kinetics early in the acute phase as immunological control mechanisms become established.

Thus, although the generally accepted constructs of dengue pathogenesis indicate that disease severity is related to/dependent upon higher plasma viremia, the available evidence is limited and inconsistent. To further explore associations between plasma viremia measured during the febrile phase and final clinical severity, we used a large database comprising detailed clinical and laboratory characterization of several thousand DENV infections among Vietnamese participants enrolled in a series of research studies over a period of 15 years.

## METHODS

### Study Population

The study population was derived from 4 prospective observational studies performed in Ho Chi Minh City, Vietnam (Supplementary Appendix 1). Briefly, studies A and B ran from 2000 to 2009 enrolling children aged 5–15 years; study A included patients with nonspecific febrile illnesses presenting to community clinics on day 1–2 of fever while study B enrolled patients admitted to a pediatric dengue ward with suspected dengue at any time during the febrile phase. Studies C (Vietnamese children aged 1–15 years, 2010–2015) and D (multicountry enrollment, children >5 years and adults, 2011–2016) enrolled outpatients presenting with possible dengue within 3 days of fever onset. The protocols for ongoing clinical assessments and blood sampling differed between the 4 studies; most notably, follow-up for participants in study C was by telephone unless the child required hospitalization. Participants in the other 3 studies were assessed daily in person. The decision to hospitalize an individual relied solely on the physician’s clinical judgment. For this analysis we selected all patients with laboratory-confirmed dengue enrolled at Vietnamese study sites.

### Dengue Diagnostics

Laboratory-confirmed dengue was defined by detection of either DENV-RNA, using reverse transcription–polymerase chain reaction (RT-PCR), or dengue NS1 antigen (Supplementary Appendix 2). The RT-PCR methodology changed over time, with a 2-step method being applied for studies A and B, and with a second, more sensitive, 1-step method applied for studies C and D. Viremia (more formally, RNAemia) was assessed on enrollment samples. Each illness episode was characterized as a probable primary or probable secondary infection based on Capture IgG results (Panbio, Australia) on paired samples.

### Study Endpoints

We selected 3 clinically relevant endpoints of interest—severe dengue, plasma leakage, and hospitalization (Supplementary Appendix 3). The definitions were based on the WHO 2009 classification [13], following standard clinical endpoint definitions for use in dengue intervention trials (Supplementary Table 1) [26]. Severe dengue comprises occurrence of severe plasma leakage, severe bleeding, and/or severe organ impairment, alone or in combination. However, these 4 studies focused on collecting detailed information on plasma leakage and bleeding, while investigations for organ dysfunction were performed at the discretion of the responsible clinician. Since the organ impairment data were opportunistic rather than systematic we concentrated on leakage and bleeding for the analysis. The plasma leakage endpoint includes all cases with either moderate or severe leakage, with definitions for both entities requiring a minimum dataset of specific information; those missing any essential criteria were classified as indeterminate plasma leakage.

### Statistical Analysis

All viremia values were transformed to the base-10 logarithm (log-10). We used logistic regression for all analyses (Supplementary Appendix 4). We used interaction terms to allow relationships between viremia and the study endpoints to differ with the following potential confounders: serotype, immune status, age, and illness day. Preliminary exploration of the data indicated no influence of sex on viremia. We allowed for nonlinear effects of log-10 viremia and age by using restricted cubic splines with knots at the 10th, 50th, and 90th percentiles [27]. Results are reported numerically as odds ratios (ORs) with 95% confidence intervals (CIs) and *P* values. We also present the effects of the various factors on the probability of occurrence of each endpoint using the parameter estimates from the logistic regression models.

**Figure 1.**
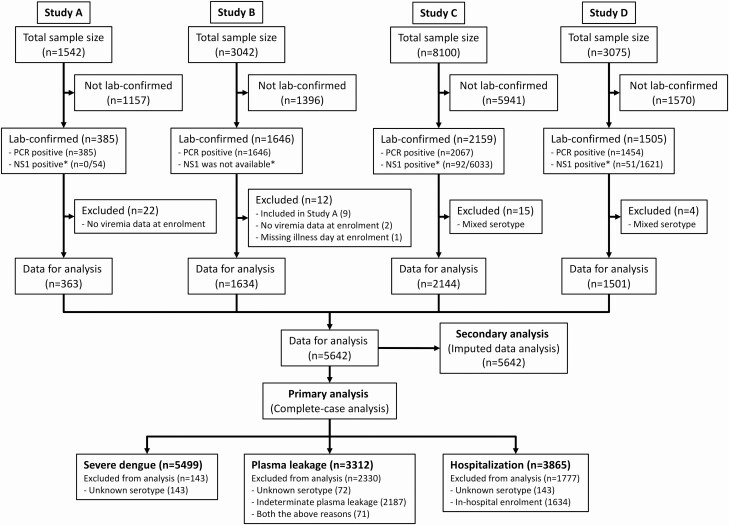
Study flow diagram. *Number testing positive for NS1 among PCR-negative cases who were tested. NS1 tests were rarely performed in study A, never done in study B, and routine in studies C and D. Abbreviations: NS1, nonstructural protein 1; PCR, polymerase chain reaction.

Since the 4 studies differed in design, the pattern of missing data varied. For all 3 endpoints, the primary analysis is a complete-case analysis (Figure 1), excluding individuals with missing serotype information. For the plasma leakage endpoint, those with indeterminate leakage status were also excluded, while for the hospitalization endpoint, study B was excluded since recruitment occurred after hospital admission. We also performed secondary analyses based on multiple imputation of missing data for unknown serotype, indeterminate plasma leakage, and indeterminate immune status, using multiple imputation by chained equation (MICE) [28, 29].

Finally, we performed sensitivity analyses to investigate whether the viremia effects differed by (1) the RT-PCR method used and (2) outpatient versus inpatient status at study enrollment. For these analyses we included each variable in the models as a main effect and with an interaction with viremia.

All analyses were performed using the statistical software R version 3.4.4 [30] and the packages “mice” and “rms” [29, 31].

## RESULTS

A total of 5686 laboratory-confirmed dengue cases were identified from the 4 studies, with 44 subsequently excluded (Figure 1). Baseline characteristics for the 5642 cases included were generally similar across the 4 studies but with some variation in line with the different study designs (Table 1). The median (first, third quartile) age was 11 (8, 14) years and male gender predominated (57.7%). Most patients (93.4%) were enrolled between illness days 2–4. All 4 serotypes are represented, but DENV-1 predominated (46.7%), while DENV-3 was infrequently identified (10.2%). In 143 cases (2.5%) the serotype was not determined. In 3224 patients (57.1%) the infection was classified as probable secondary dengue. The 1424 cases (25.2%) with indeterminate immune status were primarily participants in study C who were never hospitalized.

**Table 1.** Summary of Variables of Interest and Clinical Outcomes

	All Patients (N = 5642)	Study A (n = 363)	Study B (n = 1634)	Study C (n = 2144)	Study D (n = 1501)
Clinical variables					
Year of enrollment, range	2003–2015	2006–2008	2003–2009	2010–2014	2011–2015
Age, median (first, third quartile), years	11 (8, 14)	12 (9, 14)	12 (10, 13)	9 (6, 11)	17 (10, 26)
Male gender, n (%)	3253 (57.7)	195 (53.7)	978 (59.9)	1211 (56.5)	869 (57.9)
Illness day at enrollment, n (%)					
Day 1	415 (7.4)	59 (16.3)	8 (0.5)	66 (3.1)	282 (18.8)
Day 2	1604 (28.4)	150 (41.3)	233 (14.3)	623 (29.1)	598 (39.8)
Day 3	2309 (40.9)	114 (31.4)	606 (37.1)	968 (45.1)	621 (41.4)
Day 4	1137 (20.1)	40 (11.0)	610 (37.3)	487 (22.7)	0 (0.0)
Day 5, 6	177 (3.2)	0 (0.0)	177 (10.8)	0 (0.0)	0 (0.0)
Serotype, n (%)					
DENV-1	2637 (46.7)	233 (64.2)	946 (57.9)	826 (38.5)	632 (42.1)
DENV-2	1193 (21.1)	48 (13.2)	451 (27.6)	453 (21.1)	241 (16.1)
DENV-3	575 (10.2)	80 (22.0)	189 (11.6)	197 (9.2)	109 (7.3)
DENV-4	1094 (19.4)	2 (0.6)	48 (2.9)	576 (26.9)	468 (31.2)
Unknown	143 (2.5)	0 (0.0)	0 (0.0)	92 (4.3)	51 (3.4)
Log-10 viremia at enrollment, median (first, third quartile),^a^ copies/mL	7.3 (6.1, 8.2)	7.6 (6.6, 8.3)	7.0 (5.7, 8.1)	7.2 (6.0, 8.2)	7.5 (6.5, 8.4)
Immune status, n (%)					
Probable primary infection	994 (17.6)	134 (36.9)	290 (17.7)	254 (11.8)	316 (21.1)
Probable secondary infection	3224 (57.1)	219 (60.3)	1270 (77.7)	724 (33.8)	1011 (67.4)
Indeterminate immune status	1424 (25.2)	10 (2.8)	74 (4.5)	1166 (54.4)	174 (11.6)
Clinical outcomes, n (%)					
Severe dengue^b^	259 (4.6)	6 (1.7)	114 (7.0)	120 (5.6)	19 (1.3)
Severe vascular leakage					
+ Dengue shock syndrome	243 (4.3)	6 (1.7)	114 (7.0)	108 (5.0)	15 (1.0)
+ Respiratory distress without shock	13 (0.2)	0 (0.0)	0 (0.0)	9 (0.4)	4 (0.3)
Severe bleeding	11 (0.2)	0 (0.0)	0 (0.0)	11 (0.5)	0 (0.0)
Severe organ impairment^c^	9 (0.2)	NA	NA	9 (0.4)	0 (0.0)
Plasma leakage					
Severe	256 (4.5)	6 (1.7)	114 (7.0)	117 (5.5)	19 (1.3)
Moderate	445 (7.9)	37 (10.2)	220 (13.5)	66 (3.1)	122 (8.1)
None	2683 (47.6)	281 (77.4)	626 (38.3)	416 (19.4)	1360 (90.6)
Indeterminate	2258 (40.0)	39 (10.7)	674 (41.2)	1545 (72.1)	0 (0.0)
Hospitalization	3075 (54.5)	17 (4.7)	1634 (100.0)	965 (45.0)	459 (30.6)

Abbreviations: DENV, dengue virus; NA, not available; RT-PCR, reverse transcription–polymerase chain reaction.

^a^Calculated among the 5499 patients with positive RT-PCR.

^b^Individuals can be included in more than 1 of the 3 severe dengue categories.

^c^Laboratory investigations to detect organ involvement were not carried out in studies A or B, and were generally clinician driven rather than systematic in studies C and D.

The frequency of the 3 endpoints differed by study (Table 1), again reflecting differences in the individual study designs. Overall, 259 patients (4.6%) developed severe dengue, including 243 (4.3%) with DSS and 11 (0.2%) with severe bleeding. Severe organ impairment was diagnosed infrequently—in total, in 9 cases, all in study C. Plasma leakage was identified in 701 patients (12.4%), but 2258 patients (40.0%) could not be classified; the majority of the unclassified cases were participants in study C who were managed as outpatients but probably had minimal leakage. A total of 3075 patients (54.5%) were hospitalized, but after excluding study B this proportion fell to 40.0% (1441/4008) of all patients recruited in outpatient settings.

The 3 endpoints were observed much less frequently in the 10 064 individuals for whom dengue was not laboratory confirmed: 0.9% severe dengue, 2.6% plasma leakage, and 21.3% hospitalized (Supplementary Table 2). Although the majority of this group likely had other febrile illnesses, a few individuals may have had dengue, but by study enrollment the RT-PCR and/or NS1 tests were already negative.

### Plasma Viremia

**Figure 2.**
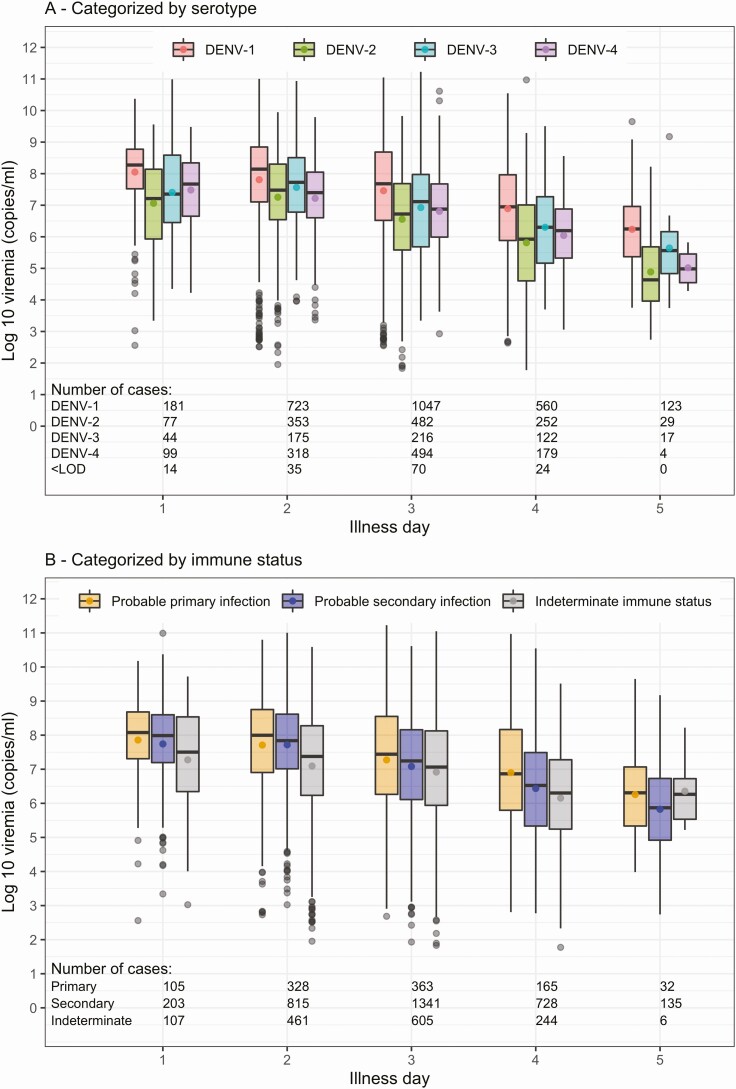
Summary of enrollment plasma viremia levels by serotype and immune status for the complete-case analysis. In each plot, plasma viremia levels are presented by illness day and colored by serotype (*A*) or immune status (*B*). The line inside each box is the median, the upper and lower margins of each box represent the interquartile range (25th–75th percentile), and the circle in each box is the mean plasma viremia level. Abbreviations: DENV, dengue virus; <LOD, under the limit of detection (not shown for panel *B* as the numbers are the same as in panel *A*).

Plasma viremia levels by illness day at enrollment, serotype, and immune status are shown in Figure 2. Viremia levels were generally lower on later days of illness, irrespective of serotype or immune status. Additionally, viremia was higher for DENV-1 compared with the other serotypes (Figure 2A). With respect to immune status, viremia appeared to be slightly lower in probable secondary compared with probable primary infection on each illness day (Figure 2B).

### Effect of Plasma Viremia on Clinical Endpoints

**Figure 3.**
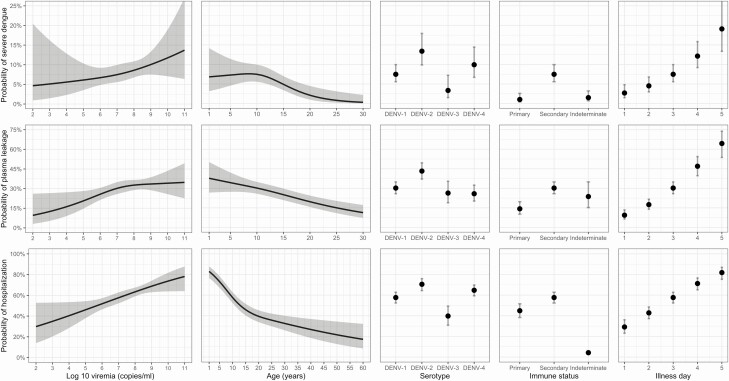
Probability of occurrence of the 3 endpoints according to each variable included in the models. Results are based on models with nonmissing data only (complete-case analysis). The estimated probability for each clinical outcome is shown with a heavy black line or dot, and the 95% confidence intervals are shown as the gray-shaded regions or by the whiskers. The probabilities are estimated for log10 viremia = 7, age = 10, illness day = 3, serotype = DENV-1, and immune status = probable secondary infection. Note that the probability of hospitalization is very low for the indeterminate immune status group because of the study design—most cases in this group come from study C, in which a second blood sample was rarely obtained from the nonhospitalized cases. Abbreviation: DENV, dengue virus.

**Figure 4.**
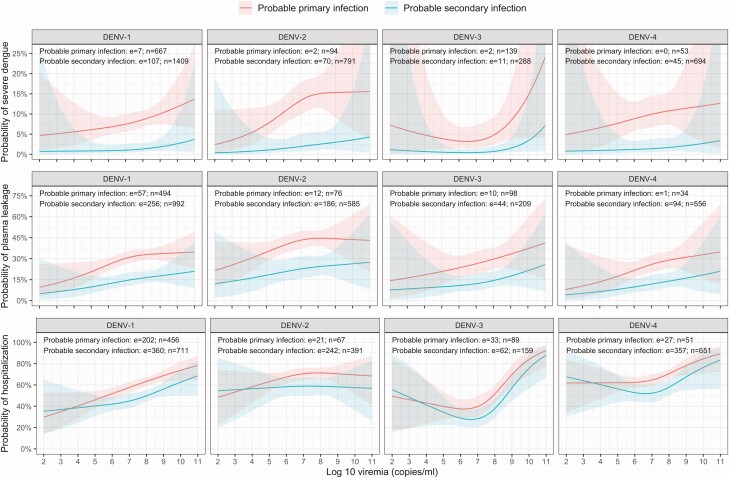
Probability of occurrence of the 3 endpoints according to plasma viremia level, by serotype and immune status. Results are based on models with nonmissing data only (complete-case analysis). The estimated probability for each clinical outcome is shown by the heavy colored lines, and the 95% confidence intervals are shown as the colored shaded regions. All probabilities are estimated for age = 10 years and illness day = 3. Abbreviations: DENV, dengue virus; e, number of events; n, number of individuals.

From the early febrile phase onwards, higher viremia increased the risk of developing all 3 endpoints—severe dengue, plasma leakage, and hospitalization—in each case with a modest but clearly apparent effect (Table 2). These effects are reasonably linear on a probability scale (Figures 3 and 4) but are nonlinear in terms of ORs so we present 2 selected contrasts, each representing a 1-log change in viremia. There was little indication of effect modification by age, serotype, immune status, or illness day for severe dengue and plasma leakage, suggesting that the effect of viremia on these endpoints is similar across the various subgroups. However, for the hospitalization endpoint, effect modification was apparent with respect to illness day.

**Table 2.** Relationships Between Variables Included in the Models and Each of the 3 Endpoints (Complete-Case Analysis)

	Severe Dengue		Plasma Leakage		Hospitalization	
Factor	OR (95% CI)	*P*	OR (95% CI)	*P*	OR (95% CI)	*P*
Log-10 viremia (copies/mL)^a,b^		.001		<.001		<.001
7 versus 6	1.13 (.88–1.43)		1.25 (1.05–1.49)		1.26 (1.09–1.47)	
8 versus 7	1.16 (.98–1.38)		1.12 (.99–1.26)		1.27 (1.12–1.44)	
All interactions of log-10 viremia		.088		.162		.003
Interaction with age	…	.042	…	.500	…	.147
Interaction with serotype	…	.730	…	.977	…	.033
Interaction with immune status	…	.379	…	.748	…	.389
Interaction with illness day	…	.087	…	.001	…	.001
Nonlinear effect of log-10 viremia	…	.007	…	.614	…	<.001
Age (years)^a,c^		<.001		<.001		<.001
10 vs 5	1.03 (.68–1.56)		.83 (.65–1.05)		.50 (.42–.59)	
15 vs 10	.64 (.48–.85)		.78 (.68–.88)		.62 (.56–.69)	
Nonlinear effect of age	…	.096	…	.633	…	<.001
Serotype^c^		.022		<.001		<.001
DENV-1	1		1		1	
DENV-2	1.91 (1.24–2.95)		1.76 (1.28–2.41)		1.76 (1.29–2.40)	
DENV-3	.43 (.18–1.00)		.83 (.52–1.31)		.48 (.32–.73)	
DENV-4	1.36 (.82–2.25)		.81 (.56–1.16)		1.35 (1.02–1.78)	
Immune status^c^		<.001		<.001		<.001
Probable primary infection	1		1		1	
Probable secondary infection	7.76 (3.00–20.08)		2.60 (1.73–3.90)		1.67 (1.24–2.25)	
Indeterminate immune status	1.47 (.45–4.79)		1.86 (.97–3.55)		.05 (.04–.08)	
Illness day at enrollment (per 1-day increase)^c^	1.71 (1.39–2.11)	<.001	2.05 (1.70–2.46)	<.001	1.82 (1.58–2.10)	<.001

Abbreviations: CI, confidence interval; DENV, dengue virus; OR, odds ratio.

^a^We allowed for nonlinear effects of log-10 viremia and age on the endpoints. To simplify interpretation of the results, ORs for 2 selected viremia and age contrasts from the models are presented.

^b^Since a number of interactions are present in the models, ORs and 95% CIs are shown for patients with age = 10, serotype = DENV-1, immune status = probable secondary infection, and illness day at enrollment = 3.

^c^Since a number of interactions are present in the models, the ORs and 95% CIs are shown for patients with log-10 viremia = 7.

In the complete-case analysis, the risk for severe dengue, plasma leakage, and hospitalization differed by serotype. The risk for all endpoints was highest for DENV-2 and lowest for DENV-3 infections. For DENV-4, the risks for severe dengue and hospitalization appeared to be borderline higher than for DENV-1 but similar to DENV-3 for plasma leakage (Table 2, Figure 3). Probable secondary infection strongly increased the risk for severe dengue compared with probable primary infection, with less marked effects for plasma leakage and hospitalization (Table 2, Figures 3 and 4). With increasing age, the risk for plasma leakage decreased almost linearly up to 30 years, while for hospitalization, there was a steeper drop from 1 to 15 years (Figure 3), likely reflecting families’/clinicians’ heightened anxieties about younger children. For severe dengue, the risk was relatively stable up to 10 years before gradually decreasing, potentially reflecting less efficient physiological compensation for leakage in the younger age group. Later illness day at enrollment was associated with an increased risk of all endpoints (Table 2, Figure 3); less-affected individuals are likely to improve more quickly and are therefore underrepresented among those recruited later.

The imputed-data analysis showed largely similar results to the complete-case analysis (Supplementary Table 3, Supplementary Figures 1 and 2). Higher plasma viremia significantly increased the risk for all 3 outcomes, with little to no effect modification by age, serotype, or immune status. In addition, patients with viremia under the limit of detection (ie, negative RT-PCR) had a low risk for all outcomes, similar to those with detectable but low-level viremia (Supplementary Figures 1 and 2).

### Sensitivity Analyses

The models including RT-PCR method or inpatient/outpatient status at enrollment showed similar effects to the main models, with higher viremia increasing the risk for all outcomes in analyses both with and without imputation of missing data (Supplementary Tables 4–7). Studies using the 1-step RT-PCR method had lower risk of plasma leakage but higher risk of hospitalization than those using the 2-step RT-PCR method, probably reflecting differences in the study populations (Supplementary Tables 4 and 5). Individuals who were inpatients at enrollment had increased risk of plasma leakage compared with outpatients (Supplementary Tables 6 and 7).

## DISCUSSION

In this pooled analysis of 5642 Vietnamese dengue cases we have clearly demonstrated that higher viremia levels during the febrile phase increase the risk for development of vascular leakage and severe dengue as well as the risk of hospitalization. Relative differences were apparent between the serotypes and in the different immune status groups, but the probability of occurrence for all 3 endpoints increased in line with log-10 plasma viremia, with generally consistent findings between the complete-case and imputed-data analyses and in the sensitivity analyses. However, the effect sizes were modest compared with the effects of a secondary immune response, and we did not identify a particular threshold associated with disease progression.

Previous efforts to explore relationships between plasma viremia and dengue severity have provided somewhat contradictory findings [14–16, 18–25]. Major strengths of this work include the very large sample size, the fact that viremia measurements were obtained early and categorized by illness day, and the rigorous endpoint classification. These characteristics, as well as the use of statistical approaches designed to address missing data and differences in study design, allowed us to establish conclusively the overarching relationship between viremia and dengue severity and to confirm that this relationship holds true for all serotypes and immune status groups.

As expected, we identified secondary immune status as a strong independent predictor of all adverse outcomes. Yet, it is notable that day-specific viremia levels were consistently lower in probable secondary than in probable primary cases, as has been reported previously [15, 17, 19, 21]. Possible explanations include the fact that tissue-sequestered virus likely influences disease pathogenesis yet is protected from detection in plasma assays. Second, in individuals with DENV immunological memory, higher viremia early in the illness evolution, particularly if boosted by ADE, is likely to elicit more robust immune responses, potentially shifting the viremia curve to the left and resulting in an earlier and/or lower virus peak. Unfortunately, however, in practice, peak viremia is rarely captured since patients seldom present before day 2–3. Interestingly, persistence of a strong effect of immune status in multivariable models that include viremia suggests that, while secondary infection may indeed influence outcomes via viremia, the relationship is complex and other pathways must also be involved.

Consistent with previous research, the study also confirms that DENV-2 carries the greatest risk of adverse outcomes despite manifesting the lowest daily viremia levels and that viremia is typically highest for DENV-1 [14, 32, 33]. However, in our dataset and in the literature generally, primary DENV-2 cases are underrepresented [15, 16, 18], which may confound the serotype analysis. The existing literature on relationships between DENV-3 (151 total cases) and DENV-4 (36 total cases) and outcome is sparse [14, 16, 20, 33], and thus the study provides confirmation that relationships with adverse outcomes are consistent across all serotypes. Interpreting the significance of between-serotype differences in the magnitude of plasma viremia by illness day is difficult, since RT-PCR measures genome copies and the relative proportions of infectious:noninfectious particles could vary between serotypes and between primary and secondary dengue.

One limitation that could affect the overall generalizability of the findings relates to the age range of the study participants. The focus of the 4 contributing studies was on pediatric dengue, and only study D enrolled adults, who formed less than 15% of the whole study population. We were careful to apply uniform definitions for the 3 endpoints across all 4 studies, but the majority of those classified as severe dengue had DSS (243/259 cases, 93.8%) and few cases with severe bleeding or severe organ impairment were identified. Age-related physiological factors increase the risk for DSS in children compared with adults, while intrinsically lower normal platelet ranges and a greater likelihood of pre-existing organ dysfunction mean that bleeding and organ impairment are more likely to occur in older age groups [34]. Thus, while the focus on outpatient recruitment of children facilitated exploration of the influence of early-stage viremia on progression to DSS, this feature also limited our capacity to capture less-common events in older populations. In addition, since laboratory tests to detect organ dysfunction were not performed systematically, the outcome data are less robust for this subcategory of severe dengue than for the more closely observed vascular leakage and bleeding categories. Most previous studies in adults have not found clear evidence of an effect of viral load on disease severity, but sample sizes were generally small with no adjustment for potential confounders [16–19, 21].

Another potential limitation involves the use of 2 different RT-PCR techniques. However, our sensitivity analysis showed consistent associations between viremia and adverse outcomes across both methods, indicating that the associations are robust. In line with this, NS1-positive but RT-PCR–negative individuals had a low risk of all outcomes, comparable to individuals with documented low-level viremia. Undoubtedly, some other study participants did have dengue but were both RT-PCR and NS1 negative and thus were not included in this analysis; however, the proportion of missed cases in this very large study is likely to be small.

We also acknowledge that retrospective pooling of data from 4 studies led to some heterogeneity and to issues with missing data for certain important variables. However, we overcame these issues through multiple imputation using the MICE method, a well-recognized system for dealing with missing data, and the similarities between the results of the complete-case and imputed-data analyses provide strong reassurance that the overall findings are robust. In real-world practice, to undertake a prospective study of this size with uniform data collection and laboratory evaluations would be extremely difficult, and in fact, the heterogeneities between the studies, taken together with our sensitivity analyses, serve to reinforce the broad generalizability of the results. Although this is the most comprehensive analysis of this nature ever undertaken, in an ideal world additional data from other ethnic groups and/or lower transmission settings where the elderly (a high-risk group missing from our dataset) remain susceptible to dengue would be used to establish conclusively that the findings are generalizable to other populations.

In conclusion, following conflicting results from less-comprehensive studies, this work provides clear evidence that, regardless of the infecting serotype or the host’s immune status, higher plasma viremia increases the risk for clinically relevant adverse outcomes associated with dengue infection. In addition to the relevance for pathogenesis studies, this knowledge could be utilized to develop risk-prediction algorithms aimed at enrolling individuals at increased risk of complications in research studies. Additionally, confirmation of a definite relationship between viremia and disease severity reinforces the rationale for targeting viremia as an endpoint in therapeutic intervention trials aimed at preventing and/or treating severe dengue.

## Supplementary Data

Supplementary materials are available at *Clinical Infectious Diseases* online. Consisting of data provided by the authors to benefit the reader, the posted materials are not copyedited and are the sole responsibility of the authors, so questions or comments should be addressed to the corresponding author.

ciaa1840_suppl_Supplementary_Materials

## Notes

***Acknowledgments.*** We thank the staff from the dengue research group at the Oxford University Clinical Research Unit for their support over many years, as well as the staff from the Hospital for Tropical Diseases and the many hospitals and clinics who recruited and followed patients in the 4 clinical studies with great dedication. We also gratefully acknowledge all the patients and their families for participating in these studies.

***Disclaimer.*** The funders played no role in the study design, data collection, analysis, interpretation or writing of the manuscript, or in the decision to submit for publication. N. L. V. and B. A. W. confirm that they had full access to all the data in the study and take personal responsibility for the decision to submit for publication.

***Financial support.*** The work was supported by the Wellcome Trust (Wellcome Trust Senior Fellowship to C. P. S. [grant number 084368/Z/07/Z]) and core grant support to the Oxford University Clinical Research Unit (grant numbers 077078/Z/05/Z, 077078/Z/05/A, 089276/B/09/Z), and by European Union grant FP7-281803 IDAMS (http://www.idams.eu/).

***Potential conflicts of interest.*** S. Y. reports receiving personal fees as a member of the Roche Advisory Board on Severe Dengue and for work on Janssen Pharmaceuticals Advisory Board for Dengue Antiviral Development. T. J. reports receiving personal fees as a member of the Roche Advisory Board on Severe Dengue. B. A. W. reports receiving personal fees for consulting and travel as a member of the Data Monitoring and Adjudication Committees for the Takeda dengue vaccine trials and as a member of the Roche Advisory Board on Severe Dengue. All other authors report no potential conflicts. All authors have submitted the ICMJE Form for Disclosure of Potential Conflicts of Interest. Conflicts that the editors consider relevant to the content of the manuscript have been disclosed.
