# Correction to: Higher Plasma Viremia in the Febrile Phase Is Associated With Adverse Dengue Outcomes Irrespective of Infecting Serotype or Host Immune Status: An Analysis of 5642 Vietnamese Cases

**DOI:** 10.1093/cid/ciad081

**Published:** 2023-02-20

An error appeared in the 15 June 2021 issue of the journal (Vuong et al. “Higher Plasma Viremia in the Febrile Phase Is Associated With Adverse Dengue Outcomes Irrespective of Infecting Serotype or Host Immune Status: An Analysis of 5642 Vietnamese Cases.” *Clin Infect Dis*; 72(12): e1074-e1083. https://doi.org/10.1093/cid/ciaa1840). In the legend for Figure 4, red is labeled as “Probable primary infection” and blue is labeled as “Probable secondary infection,” although it should be the other way around.

The authors regret the error.

