# Supplementary material for: Higher Plasma Viremia in the Febrile Phase Is Associated With Adverse Dengue Outcomes Irrespective of Infecting Serotype or Host Immune Status: An Analysis of 5642 Vietnamese Cases
Source: Clin Infect Dis. 2020 Dec 19;72(12):e1074–83. doi: 10.1093/cid/ciaa1840 (PMC8204785; doi:10.1093/cid/ciaa1840)
Supplement: ciaa1840_suppl_Supplementary_Materials [file ciaa1840_suppl_supplementary_materials.docx]

**Appendix 1. Protocol synopses for the four studies**

The four studies described below were performed as part of the longstanding collaboration between the Oxford University Clinical Research Unit and the Hospital for Tropical Diseases in Ho Chi Minh City, Vietnam. All studies were approved by the Scientific and Ethics Committee of the hospital and by the Oxford Tropical Research Ethics Committee.

**Study A**

Title: A study of early dengue disease among children in the community

Study objectives: this study was part of a larger programme of work aiming to a) estimate the burden of paediatric dengue disease in HCMC, b) explore the mechanisms of the endothelial dysfunction associated with dengue by detailed study of well characterized patients throughout the evolution of the disease and c) investigate whether commonly available laboratory parameters (haematocrit, platelets, urine protein) measured during the febrile period can be used to predict subsequent disease severity.

Study period: 2000-2009

Study subjects: previously healthy children aged 5-15 years, presenting to hospital outpatient clinics or to one of several local health centers in Ho Chi Minh City on day 1 or 2 of a non-specific febrile illness. Children were followed daily in the community. In the event of admission to hospital, patients continued to be followed either on the dengue ward or on PICU of the Hospital for Tropical Diseases in HCMC, according to clinical severity.

Study setting: parents/guardians of eligible patients were informed about the study and gave consent. After enrollment baseline blood and urine samples were obtained. Patients were then asked to return each day for 7 days or until full recovery, and then at 1-2 weeks and 1-2 months for follow up. The study nurse assessed each child in the clinic every day, obtained daily blood and urine samples, and requested a medical opinion if there were any concerns about the child or the daily blood results. Two trained study doctors supervised the nurses in the clinics and followed any patients admitted to the hospital. Management in the clinic or referral for hospital admission was at the discretion of the clinic doctors, in consultation with the dengue study doctors.

Data collection: demographic and clinical data were collected on each patient at study entry using a standard case report form. Clinical progress was documented each day specifically focusing on the occurrence of bleeding manifestations or signs of vascular leakage. There was no systematic data collection regarding organ dysfunction as the clinicians felt that these problems were rare in this age group, and no biochemistry (eg liver/renal function) test results were documented in the research files.

Laboratory confirmation of dengue: either positive reverse transcription polymerase chain reaction (RT-PCR) or seroconversion by ELISA (IgM or IgG or both). Testing for dengue non-structural protein 1 (NS1) was performed occasionally but was not part of the original study protocol.

Publications:

1. Dung NT, Duyen HT, Thuy NT, et al. Timing of CD8+ T cell responses in relation to commencement of capillary leakage in children with dengue. *J Immunol* 2010; **184**(12):7281-7287
2. Duyen HT, Ngoc TV, Ha do T, et al. Kinetics of plasma viremia and soluble nonstructural protein 1 concentrations in dengue: differential effects according to serotype and immune status. *J Infect Dis* 2011; **203**(9):1292-1300
3. Hanh Tien NT, Lam PK, Duyen HT, et al. Assessment of microalbuminuria for early diagnosis and risk prediction in dengue infections. *PLoS One* 2013; **8**(1):e54538

**Study B**

Title: Mild Dengue Study

Study objectives: this study was part of a larger programme of work aiming to a) estimate the burden of paediatric dengue disease in HCMC, b) explore the mechanisms of the endothelial dysfunction associated with dengue by detailed study of well characterized patients throughout the evolution of the disease and c) investigate whether commonly available laboratory parameters measured during the febrile period can be used to predict subsequent disease severity.

Study period: 2000-2009

Study subjects: children 5-15 years presenting to the Hospital for Tropical Diseases in HCMC with a febrile illness consistent with possible dengue, who required hospital admission to the paediatric dengue wad, NOT those admitted to HDU/ICU. Only children admitted from home were included (i.e. not transfers from other hospitals).

Study setting: parents/guardians of eligible patients were informed about the study and gave consent. All patients were followed daily until discharge with simple study notes and a daily full blood count (plus any other tests clinically indicated), and treated according to individual diagnoses. On the day of study enrolment all patients had a plasma sample obtained for research investigations. On the 6^th^ illness day a second plasma sample was obtained. At discharge the notes were reviewed and each child was assigned a final diagnosis and clinical disease category, using carefully defined severity criteria for vascular leak and bleeding. The patients were asked to return for follow up at one month, and again at two months if there were any ongoing concerns.

Data collection: demographic and clinical data were collected on each patient at study entry using a standard case report form. Clinical progress was documented each day specifically focusing on the occurrence of bleeding manifestations or signs of vascular leakage. There was no systematic data collection regarding organ dysfunction as the clinicians felt that these problems were rare in this age group, and no biochemistry (eg liver/renal function) test results were documented in the research files.

Laboratory confirmation of dengue: either positive RT-PCR or seroconversion by ELISA (IgM or IgG or both). NS1 testing was not performed.

Publications:

1. Dung NT, Duyen HT, Thuy NT, et al. Timing of CD8+ T cell responses in relation to commencement of capillary leakage in children with dengue. *J Immunol* 2010; **184**(12):7281-7287
2. Trung DT, Thao LTT, Dung NM, et al. Clinical Features of Dengue in a Large Vietnamese Cohort: Intrinsically Lower Platelet Counts and Greater Risk for Bleeding in Adults Than Children. *PLoS Negl Trop Dis* 2012; **6**(6):e1679.
3. Lam PK, Ngoc TV, Thuy TT, et al*.* The value of daily platelet counts for predicting dengue shock syndrome: Results from a prospective observational study of 2301 Vietnamese children with dengue. *PLoS Negl Trop Dis* 2017; **11**(4): e0005498
4. Hoang Quoc C, Henrik S, Isabel RB, et al. Synchrony of Dengue Incidence in Ho Chi Minh City and Bangkok. *PLoS Negl Trop Dis* 2016; **10**(12):e0005188

**Study C**

Title: Laboratory diagnosis and prognosis of severe dengue (ClinicalTrials.gov Identifier: NCT01421732)

Study objectives: the aims of this study were to

- Define the diagnostic accuracy of early NS1 detection in severe dengue
- Develop a prognostic algorithm for the early identification of severe dengue cases
- Discover and evaluate new early biomarkers of severe dengue
- Understand the phylogeography of DENV in the super-urban setting of Ho Chi Minh City

Study period: 2010-2015

Study subjects: patients 1-15 years of age, presenting to outpatient departments of the Hospital for Tropical Diseases, Children’s Hospital Number 1, or Children’s Hospital Number 2 (HCMC) with fever at presentation (or history of fever) and less than 72 hours of symptom history, and a clinical suspicion of dengue.

Study setting: After informed consent by a parent or guardian all participants had a venous blood sample obtained at study enrolment. Standard clinical care and laboratory monitoring continued as normal, with daily or alternate days visits to ambulatory care at the outpatient department as directed by the treating clinician. Parents/guardians of patients who did not require hospitalization were contacted by a research nurse each day for six consecutive days after enrolment or until the child was afebrile for 48 hours and had returned to normal routines; the nurse completed a simple telephone questionnaire at each contact. 10% of the non-hospitalized patients were randomly chosen to collect a convalescent blood sample on or after day six of illness for the purposes of determining immune status. In case of hospitalization, all patients were followed up daily until discharge by study staff, and a blood sample was routinely collected before discharge for the immune status diagnosis.

Data collection: demographic, clinical, and laboratory data were collected using an electronic case record form. Among hospitalized cases only, detailed information on vascular leakage and bleeding was collected, together with results of laboratory tests and other investigations carried out if organ dysfunction was clinically suspected.

Laboratory confirmation of dengue: either detection of DENV RNA by molecular methods, culture of infectious virus, positive NS1, or serology (IgM seroconversion).

Publications:

1. Tuan NM, Nhan HT, Chau NVV, et al. Sensitivity and Specificity of a Novel Classifier for the Early Diagnosis of Dengue. *PLoS Negl Trop Dis* 2015; **9**(4): e0003638
2. Tuan NM, Nhan HT, Chau NVV, et al. An Evidence-Based Algorithm for Early Prognosis of Severe Dengue in the Outpatient Setting. *Clin Infect Dis* 2017; **64**(5): 656-663
3. Quyen NTH, Kien DTH, Rabaa M, et al. Chikungunya and Zika Virus Cases Detected against a Backdrop of Endemic Dengue Transmission in Vietnam. *Am J Trop Med Hyg* 2017; **97**(1):146-150
4. Whitehorn J, Kien DTH, Quyen NTH, et al. Genetic variants of MICB and PLCE1 and associations with the laboratory features of dengue. *BMC Infect Dis* 2017; **17**(1):412

**Study D**

Title: IDAMS – observational study in early dengue (ClinicalTrials.gov Identifier: NCT01550016)

Study objectives: to improve diagnosis and clinical management of dengue through approaches designed a) to differentiate between dengue and other common febrile illness within 72 h of fever onset, and b) among patients with dengue to identify markers predictive of the likelihood of evolving to a more severe disease course.

Study period: 2011-2016

Study subjects: both adults and children (≥5 years) were eligible for enrolment. This was a prospective multi-centre observational study that enrolled approximately 7,500 patients presenting with a febrile illness consistent with possible dengue to outpatient health facilities in urban centres in eight countries across Asia and Latin America. Following appropriate informed consent, subjects presenting at one of the designated sites with fever for ≤ 72 hours without localizing features, i.e. consistent with a possible diagnosis of dengue, were enrolled.

Study setting: Following enrolment, clinical history and examination findings were recorded in the case report form and a 3–5 ml (age-dependent) research blood sample was obtained, together with appropriate samples to measure a range of haematological and biochemical parameters in line with local laboratory capacity. Patients were then reviewed daily in the OPD until fully recovered and afebrile for 24 hours, or for up to six days from enrolment. A full blood count was carried out each day, and on the last acute illness visit (within approximately 24 hours of defervescence) a second sample for a biochemical profile was obtained together with a sample for serology. All patients were then asked to attend a final follow-up visit around day 10-14 of illness, at least one week from the last visit during the acute illness. All management decisions throughout the acute illness were at the discretion of the clinic physicians. Any patient requiring hospital admission continued to be followed daily using a similar but more detailed CRF, with the indication(s) for admission documented, and all management interventions recorded together with the physician’s rationale for these interventions.

Data collection: a structured clinical questionnaire was completed upon enrolment and then once daily for up to six days for all patients in the study. This CRF included detailed clinical signs and symptoms, as well as all standard laboratory results including liver and renal function.

Laboratory confirmation of dengue: any case with virological evidence of dengue as shown by a positive RT-PCR assay or NS1 ELISA test, was defined as having laboratory-confirmed dengue.

Publications:

1. Nguyet MN, Duong TH, Trung VT, et al. Host and viral features of human dengue cases shape the population of infected and infectious Aedes aegypti mosquitoes. *Proc Natl Acad Sci USA* 2013; **110**(22):9072-9077
2. Jaenisch T, Tam DTH, Kieu NTT, et al. Clinical evaluation of dengue and identification of risk factors for severe disease: protocol for a multicentre study in 8 countries. *BMC Infect Dis* 2016; **16**: 120
3. Vuong NL, Le Duyen HT, Lam PK, et al. C-reactive protein as a potential biomarker for disease progression in dengue: a multi-country observational study. *BMC Med* 2020; 18(1): 35

**Appendix 2. Definitions used for the dengue diagnostics**

The criteria for laboratory confirmed dengue used in the four studies were harmonized for this pooled data analysis. Only individuals with positive RT-PCR or NS1 antigen testing on plasma samples obtained at study enrolment are included here. In studies A and B, plasma viremia levels were measured using an internally controlled, serotype-specific, real-time, two-step RT-PCR assay, following the method described previously.[1] In studies C and D, plasma viremia levels were measured by one-step RT-PCR using a validated assay.[2] In all studies NS1 antigen detection was performed using Platelia NS1 kits (Biorad), while serological assays were performed using IgG Capture ELISA Kits (Panbio, Australia), both according to the manufacturer’s instructions. All diagnostic tests were carried out in batches at intervals but no results were available in real time to the physicians providing clinical care.

**Limit of Detection (LOD) for plasma viremia**

There was no formal validation of the LOD for plasma viremia for the two-step RT-PCR used in studies that were conducted during the early years (studies A and B).

In the one-step RT-PCR used in studies C and D the LOD was: 5 copies/ml for DENV-1 and DENV-3; 1 copy/ml for DENV-2; and 10 copies/ml for DENV-4.[2]

**Illness phases**

Illness day: number of days since symptom onset (normally fever). Day 1 is the day of symptom onset.

Febrile phase: illness days 1 to 5.

Convalescent phase: illness days 6 to 10.

**Immune Status**

Probable primary infection: a probable primary infection was defined by two negative/equivocal dengue-specific Capture IgG results on two consecutive specimens taken at least 2 days apart during the febrile or convalescent phases, with at least one specimen obtained during the convalescent phase. Of note, several patients without specimens between illness days 6-10, but in whom the IgG response was negative/equivocal after illness day 10, were considered to have had negative/equivocal IgG responses during the convalescent phase.

A probable secondary infection was defined by a positive dengue-specific IgG identified during either or both the febrile and convalescent phases.

In all other cases immune status was classified as indeterminate.

**References**

1. Simmons CP, Popper S, Dolocek C, et al. Patterns of host genome-wide gene transcript abundance in the peripheral blood of patients with acute dengue hemorrhagic fever. J Infect Dis 2007; 195(8): 1097-107.
2. Hue KD, Tuan TV, Thi HT, et al. Validation of an internally controlled one-step real-time multiplex RT-PCR assay for the detection and quantitation of dengue virus RNA in plasma. *J Virol Methods* 2011; 177(2): 168-73.

**Appendix 3. Clinical severity classification for dengue infected patients**

**Table S1. Clinical severity definitions, in line with the 2009 World Health Organization classification^1^**

| **Outcome** | **Definition** |
| --- | --- |
| Severe dengue | One or more of the following:   1. Severe plasma leakage resulting in dengue shock syndrome and/or respiratory distress due to fluid accumulation 2. Severe bleeding 3. Severe organ impairment |
|  | Dengue Shock Syndrome: Pulse pressure (difference between systolic and diastolic pressures) ≤ 20 mmHg or hypotension for age plus signs of poor capillary perfusion (cold extremities, delayed capillary refill, or rapid pulse rate). Clinician’s assessment was accepted for occasional cases where the PP was between 20-25. |
|  | Respiratory Distress: Increased respiratory rate for age, with signs of increased work of breathing (retractions, nasal flaring, accessory muscle use) and need for additional support such as oxygen supplementation, CPAP or intubation |
| Plasma leakage | 1. Haemoconcentration. Defined as > 20% increase in hematocrit (HCT) from baseline (minimum HCT within illness days 1-3) to acute phase (maximum HCT within illness days 4-7) 2. And/or evidence of fluid accumulation (pleural/peritoneal) on ultrasound or x-ray |
|  | Severe: Dengue shock syndrome and/or respiratory distress due to plasma leakage |
|  | Moderate: Evidence of plasma leakage but never developed shock or respiratory distress |
|  | None: Haemoconcentration < 20% (definition as above) and no evidence of fluid accumulation if CXR/USS done |
|  | Indeterminate: Missing data for HCT (either baseline or acute phase or both) |
| Severe bleeding | One or more of the following:   1. Any bleeding leading to hemodynamic instability 2. Any bleeding resulting in death or permanent disability 3. Any bleeding into a critical organ (e.g., central nervous system bleed) 4. Any bleeding that results in need for blood transfusion 5. Any bleeding that persists after measures are taken to stop bleeding (e.g., application of pressure) AND patient requires more intensive monitoring in an ICU or HDU |
| Severe organ impairment | Assessments were not systematic. For patients investigated on the basis of the attending clinician’s concerns the following definitions were applied. |
|  | Severe liver involvement: An acute clinical syndrome consistent with acute hepatitis, with new onset jaundice  or coagulopathy (INR>1.5) or encephalopathy. |
|  | Severe neurological involvement: Any new onset acute neurological signs or symptoms, except occurrence of a single simple febrile convulsion with full recovery within 30 minutes. |
|  | Severe renal involvement: Increase in creatinine to more than 1.5 times the upper limit of normal for age, without existing kidney disease. |

**References**

1. World Health Organization. Dengue: guidelines for diagnosis, treatment, prevention and control: World Health Organization; 2009.

**Appendix 4. Treatment of missing data**

We used Multiple Imputation by Chained Equations (MICE) as implemented in the R package ‘mice’ version 2.30^1^ to generate multiple imputed data sets based on a set of imputation models; each variable with missing values has one imputation model. The chosen imputation models were a) polytomous logistic regression for serotype and b) logistic regression for immune status and plasma leakage. As recommended^2^, we included all available baseline data (study, age, gender, weight, enrolment date [as days since 01 January 2000], illness day at enrolment, serotype, immune status, HCT and platelet count at enrolment) and the outcomes (hospitalization along with its characteristics [illness day when hospitalized and length of hospital stay], plasma leakage, and severe dengue) in the imputation models. We did not include nonlinear terms or interactions in any of the imputation models. In total 20 imputed datasets were created and 25 cycles per dataset were performed. We performed one imputation procedure to analyze the severe dengue and plasma leakage outcomes, and another imputation procedure to analyze the hospitalization outcome because all the patients enrolled in study B were excluded from this outcome analysis. All imputation models converged when we checked the convergence plots.

For viremia, since none of the measured values were below 35 copies/ml, all viremia values lower than the LOD were set to 1 copy/ml (meaning value zero on the log-10 scale) and a dummy binary variable (Yes/No) was created to describe whether the viremia result was lower than the LOD or not.

**References**

1. van Buuren S, Groothuis-Oudshoorn K. mice: Multivariate Imputation by Chained Equations in R. *Journal of Statistical Software* 2011; **45**(3): 1-67.
2. White IR, Royston P, Wood AM. Multiple imputation using chained equations: Issues and guidance for practice. *Statistics in medicine* 2011; **30**(4): 377-99.

**Appendix 5. Characteristics of patients for whom dengue was not lab-confirmed**

**Table S2. Summary of important variables and clinical outcomes among patients for whom dengue was not lab-confirmed**

|  | All patients  (N=10064) | Study A  (N=1157) | Study B  (N=1396) | Study C  (N=5941) | Study D  (N=1570) |
| --- | --- | --- | --- | --- | --- |
|  |  |  |  |  |  |
| Age (year), *mean (SD)* | 9.2 (7.4) | 9.9 (2.6) | 11.0 (2.4) | 6.1 (3.4) | 18.8 (13.0) |
| Gender male, *n (%)* | 5746 (57.1) | 676 (58.4) | 810 (58.0) | 3337 (56.2) | 923 (58.8) |
| Illness day at enrolment, *n (%)* |  |  |  |  |  |
| - Day 1 | 1059 (10.5) | 210 (18.2) | 13 (0.9) | 392 (6.6) | 444 (28.3) |
| - Day 2 | 3635 (36.1) | 749 (64.7) | 246 (17.6) | 1947 (32.8) | 693 (44.1) |
| - Day 3 | 3828 (38.0) | 142 (12.3) | 480 (34.4) | 2775 (46.7) | 431 (27.5) |
| - Day 4 | 1404 (14.0) | 51 (4.4) | 524 (37.5) | 827 (13.9) | 2 (0.1) |
| - Day 5, 6, 7 | 138 (1.4) | 5 (0.4) | 133 (10.3) | 0 (0.0) | 0 (0.0) |
| Severe disease*, *n (%)* | 90 (0.9) | 11 (1.0) | 66 (4.7) | 11 (0.2) | 2 (0.1) |
| - Severe vascular leakage |  |  |  |  |  |
| + Dengue shock syndrome | 79 (0.8) | 11 (1.0) | 62 (4.4) | 5 (0.1) | 1 (0.1) |
| + Respiratory distress without shock | 2 (0.0) | 0 (0.0) | 0 (0.0) | 2 (0.0) | 0 (0.0) |
| - Severe organ impairment^&^ | 11 (0.1) | NA | 3 (0.2) | 7 (0.1) | 1 (0.1) |
| - Severe bleeding | 3 (0.0) | 0 (0.0) | 2 (0.1) | 1 (0.0) | 0 (0.0) |
| Plasma leakage, *n (%)* |  |  |  |  |  |
| - Severe | 81 (0.8) | 11 (1.0) | 62 (4.4) | 7 (0.1) | 1 (0.1) |
| - Moderate | 178 (1.8) | 3 (0.3) | 161 (11.5) | 3 (0.1) | 11 (0.7) |
| - None | 2371 (23.6) | 2 (0.2) | 607 (43.5) | 204 (3.4) | 1558 (99.2) |
| - Indeterminate | 7434 (73.9) | 1141 (98.6) | 566 (40.5) | 5727 (96.4) | 0 (0.0) |
| Hospitalization, *n (%)* | 2148 (21.3) | 43 (3.7) | 1396 (100.0) | 594 (10.0) | 115 (7.3) |

*All outcomes are according to the definitions used for the confirmed dengue patients; *Individuals can be included in more than one of the three severe dengue categories; ^&^Laboratory investigations to detect organ involvement were not carried out in Studies A or B, and were clinician driven rather than systematic in Studies C and D*

*NA: not available; SD: standard deviation.*

**Appendix 6. Results for the secondary analyses using imputed data**

**Table S3. Relationships between variables included in the models and each of the three endpoints after imputation of missing data**

|  | **Severe dengue** |  | **Plasma leakage** |  | **Hospitalization** |  |
| --- | --- | --- | --- | --- | --- | --- |
| **Factor** | **OR (95% CI)** | ***P*** | **OR (95% CI)** | ***P*** | **OR (95% CI)** | ***P*** |
| Log 10 viremia (copies/ml)^1^* |  | 0.001 |  | <0.001 |  | <0.001 |
| - 7 versus 6 | 1.22 (0.98 - 1.52) |  | 1.23 (1.08 - 1.39) |  | 1.26 (1.15 - 1.38) |  |
| - 8 versus 7 | 1.16 (0.97 - 1.37) |  | 1.19 (1.06 - 1.34) |  | 1.25 (1.12 - 1.40) |  |
| All interactions of log 10 viremia | - | 0.548 | - | 0.994 | - | 0.003 |
| - Interaction with age |  | 0.165 |  | 0.961 |  | 0.085 |
| - Interaction with serotype |  | 0.908 |  | 0.994 |  | 0.424 |
| - Interaction with immune status |  | 0.782 |  | 0.966 |  | 0.177 |
| - Interaction with illness day |  | 0.186 |  | 0.389 |  | 0.001 |
| Non-linear effect of log 10 viremia | - | 0.176 | - | 0.877 | - | <0.001 |
| Age (years)^2^* |  | <0.001 |  | <0.001 |  | <0.001 |
| - 10 versus 5 | 1.24 (0.84 - 1.85) |  | 1.09 (0.89 - 1.34) |  | 0.94 (0.82 - 1.07) |  |
| - 15 versus 10 | 0.65 (0.48 - 0.88) |  | 0.89 (0.80 - 1.00) |  | 0.91 (0.84 - 0.98) |  |
| Non-linear effect of age | - | 0.017 | - | 0.007 | - | 0.030 |
| Serotype^2^ |  | 0.091 |  | <0.001 |  | 0.037 |
| - DENV-1 | 1 |  | 1 |  | 1 |  |
| - DENV-2 | 1.66 (1.08 - 2.55) |  | 1.52 (1.14 - 2.04) |  | 1.18 (0.92 - 1.52) |  |
| - DENV-3 | 0.43 (0.19 - 0.96) |  | 0.89 (0.56 - 1.41) |  | 0.56 (0.39 - 0.80) |  |
| - DENV-4 | 1.09 (0.67 - 1.76) |  | 0.68 (0.50 - 0.93) |  | 1.04 (0.82 - 1.32) |  |
| Immune status^2^ |  | <0.001 |  | <0.001 |  | <0.001 |
| - Probable primary infection | 1 |  | 1 |  | 1 |  |
| - Probable secondary infection | 8.26 (3.41 - 20.03) |  | 2.58 (1.80 - 3.70) |  | 2.13 (1.61 - 2.82) |  |
| Illness day at enrolment (per one day increase)^2^ | 1.70 (1.39 - 2.07) | <0.001 | 1.30 (1.10 - 1.52) | 0.014 | 1.53 (1.37 - 1.72) | <0.001 |

**We allowed for non-linear effects of log-10 viremia and age on the endpoints. To simplify interpretation of the results, ORs for two selected viremia and age contrasts from the models are presented.*

*^1^Since a number of interactions are present in the models, ORs and 95% CIs are shown for patients with age = 10, serotype = DENV-1, immune status = probable secondary infection, and illness day at enrolment = 3.*

*^2^Since a number of interactions are present in the models, the ORs and 95% CIs are shown for patients with log 10 viremia = 7.*

*CI: confidence interval; OR: odds ratio*

**Figure S1. Probability of occurrence of the 3 endpoints according to each variable after imputation of missing data**


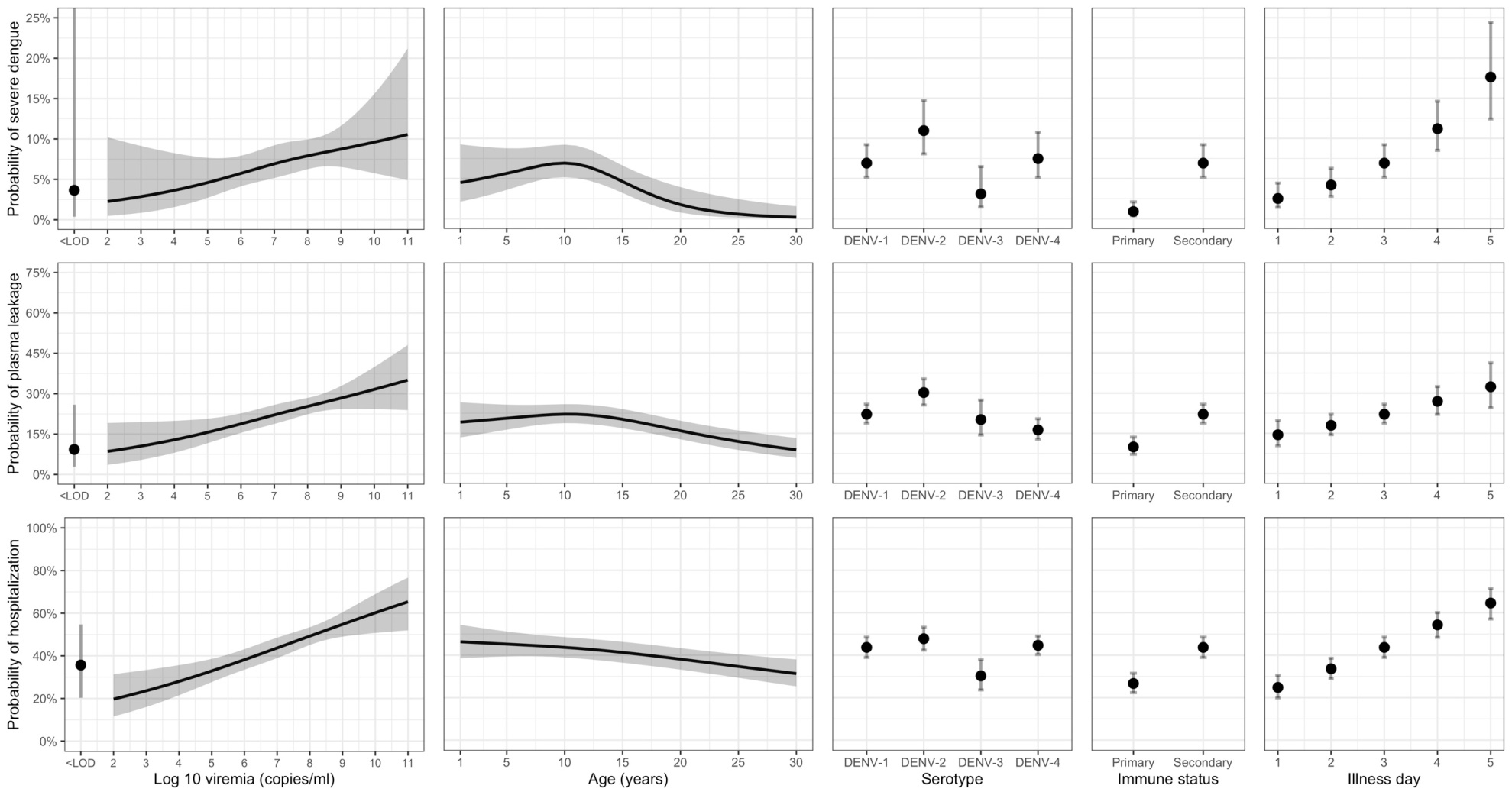


*Results are based on models after imputation of missing data. The estimated probability for each clinical outcome is shown by a black line or a black dot, and the 95% confidence intervals are shown as shaded grey regions or by the whiskers. The probabilities are estimated for: log 10 viremia = 7; age = 10; illness day = 3; serotype = DENV-1; and immune status = probable secondary infection. <LOD: lower than the limit of detection.*

**Figure S2. Probability of occurrence of the 3 endpoints according to plasma viremia level, by serotype and immune status after imputation of missing data.**


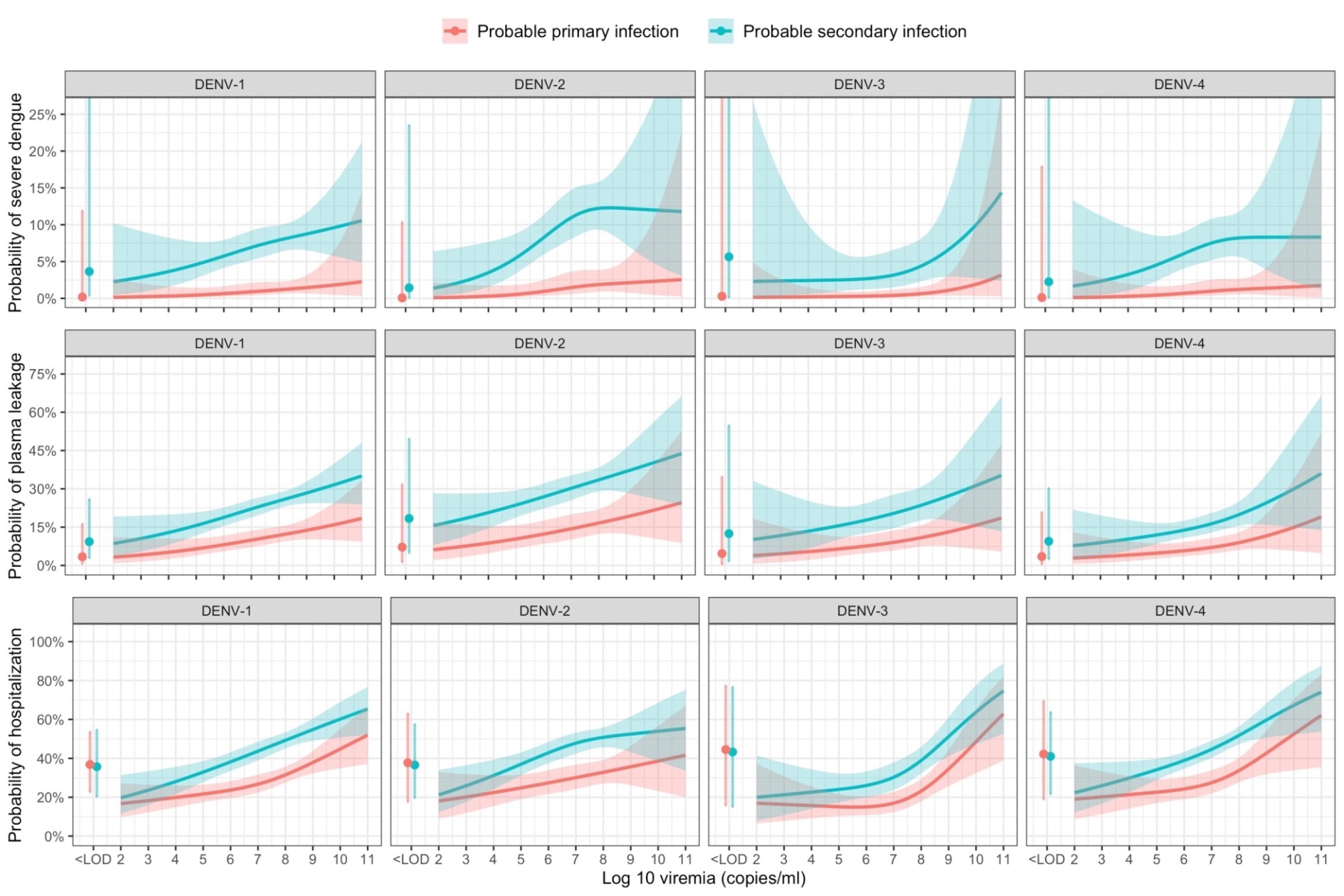


*The estimated probability for each clinical outcome is shown by coloured lines with the 95% confidence intervals shown as shaded coloured regions. All figures are shown for age 10 years and illness day 3. <LOD: lower than the limit of detection.*

**Appendix 7. Results for the sensitivity analyses for RT-PCR method**

**Table S4. Relationships between variables and each of the three endpoints – sensitivity analysis including PCR method (complete case analysis)**

|  | **Severe dengue** |  | **Plasma leakage** |  | **Hospitalization** |  |
| --- | --- | --- | --- | --- | --- | --- |
| **Factor** | **OR (95% CI)** | ***P*** | **OR (95% CI)** | ***P*** | **OR (95% CI)** | ***P*** |
| Log 10 viremia (copies/ml)^1^* |  | 0.002 |  | <0.001 |  | <0.001 |
| - 7 versus 6 | 1.13 (0.83 - 1.53) |  | 1.21 (0.96 - 1.52) |  | 1.29 (1.09 - 1.53) |  |
| - 8 versus 7 | 1.04 (0.82 - 1.33) |  | 1.18 (1.00 - 1.38) |  | 1.19 (1.04 - 1.38) |  |
| All interactions of log 10 viremia | - | 0.137 | - | 0.134 | - | 0.009 |
| Nonlinear effect of log 10 viremia | - | 0.014 | - | 0.478 | - | 0.012 |
| Age (years)^2^* |  | <0.001 |  | <0.001 |  | <0.001 |
| - 10 versus 5 | 1.17 (0.75 - 1.81) |  | 0.71 (0.55 - 0.92) |  | 0.54 (0.45 - 0.65) |  |
| - 15 versus 10 | 0.65 (0.49 - 0.87) |  | 0.75 (0.66 - 0.86) |  | 0.63 (0.56 - 0.69) |  |
| Non-linear effect of age | - | 0.041 | - | 0.762 | - | 0.002 |
| Serotype^2^ |  | 0.035 |  | <0.001 |  | 0.062 |
| - DENV-1 | 1 |  | 1 |  | 1 |  |
| - DENV-2 | 1.83 (1.18 - 2.84) |  | 1.74 (1.26 - 2.39) |  | 1.45 (1.03 - 2.03) |  |
| - DENV-3 | 0.43 (0.18 - 1.02) |  | 0.75 (0.47 - 1.18) |  | 0.74 (0.47 - 1.19) |  |
| - DENV-4 | 1.10 (0.64 - 1.90) |  | 1.08 (0.73 - 1.61) |  | 0.85 (0.63 - 1.15) |  |
| Immune status^2^ |  | <0.001 |  | <0.001 |  | <0.001 |
| - Probable primary infection | 1 |  | 1 |  | 1 |  |
| - Probable secondary infection | 8.02 (3.07 - 20.9) |  | 2.60 (1.73 - 3.92) |  | 1.63 (1.18 - 2.26) |  |
| - Indeterminate immune status | 1.35 (0.41 - 4.44) |  | 1.92 (1.00 - 3.69) |  | 0.04 (0.02 - 0.06) |  |
| Illness day at enrolment (per one day increase)^2^ | 1.81 (1.45 - 2.25) | <0.001 | 1.94 (1.61 - 2.34) | <0.001 | 1.84 (1.57 - 2.14) | <0.001 |
| PCR method (one-step versus two-step)^2^ | 1.54 (0.99 - 2.42) | 0.279 | 0.52 (0.38 - 0.70) | <0.001 | 72.31 (24.24 - 215.71) | <0.001 |

**We allowed for non-linear effects of log-10 viremia and age on the endpoints. To simplify interpretation of the results, ORs for two selected viremia and age contrasts from the models are presented.*

*^1^Since a number of interactions are present in the models, ORs and 95% CIs are shown for patients with age = 10, serotype = DENV-1, immune status = probable secondary infection, and illness day at enrolment = 3.*

*^2^Since a number of interactions are present in the models, the ORs and 95% CIs are shown for patients with log 10 viremia = 7.*

*CI: confidence interval; OR: odds ratio*

**Table S5. Relationships between variables and each of the three endpoints – sensitivity analysis including PCR method (imputed data analysis)**

|  | **Severe dengue** |  | **Plasma leakage** |  | **Hospitalization** |  |
| --- | --- | --- | --- | --- | --- | --- |
| **Factor** | **OR (95% CI)** | ***P*** | **OR (95% CI)** | ***P*** | **OR (95% CI)** | ***P*** |
| Log 10 viremia (copies/ml)^1^* |  | 0.002 |  | <0.001 |  | <0.001 |
| - 7 versus 6 | 1.24 (0.94 - 1.62) |  | 1.29 (1.10 - 1.50) |  | 1.28 (1.16 - 1.40) |  |
| - 8 versus 7 | 1.05 (0.82 - 1.33) |  | 1.29 (1.11 - 1.51) |  | 1.20 (1.06 - 1.34) |  |
| All interactions of log 10 viremia | - | 0.571 | - | 0.967 | - | 0.004 |
| Nonlinear effect of log 10 viremia | - | 0.171 | - | 0.888 | - | 0.004 |
| Age (years)^2^* |  | <0.001 |  | 0.001 |  | <0.001 |
| - 10 versus 5 | 1.28 (0.85 - 1.95) |  | 0.87 (0.71 - 1.08) |  | 1.05 (0.91 - 1.20) |  |
| - 15 versus 10 | 0.66 (0.49 - 0.89) |  | 0.84 (0.75 - 0.94) |  | 0.94 (0.87 - 1.02) |  |
| Non-linear effect of age | - | 0.017 | - | 0.772 | - | 0.002 |
| Serotype^2^ |  | 0.095 |  | 0.003 |  | 0.413 |
| - DENV-1 | 1 |  | 1 |  | 1 |  |
| - DENV-2 | 1.63 (1.06 - 2.53) |  | 1.66 (1.24 - 2.23) |  | 0.99 (0.76 - 1.29) |  |
| - DENV-3 | 0.42 (0.19 - 0.95) |  | 0.83 (0.52 - 1.34) |  | 0.67 (0.46 - 0.97) |  |
| - DENV-4 | 1.03 (0.61 - 1.74) |  | 1.04 (0.75 - 1.45) |  | 0.78 (0.61 - 0.99) |  |
| Immune status^2^ |  | <0.001 |  | <0.001 |  | <0.001 |
| - Probable primary infection | 1 |  | 1 |  | 1 |  |
| - Probable secondary infection | 8.37 (3.43 - 20.39) |  | 2.34 (1.62 - 3.36) |  | 2.30 (1.70 - 3.12) |  |
| Illness day at enrolment (per one day increase)^2^ | 1.74 (1.41 - 2.14) | <0.001 | 1.17 (1.00 - 1.38) | 0.263 | 1.50 (1.33 - 1.68) | <0.001 |
| PCR method (one-step versus two-step)^2^ | 1.17 (0.75 - 1.82) | 0.566 | 0.43 (0.33 - 0.57) | <0.001 | 30.54 (10.60 - 87.99) | <0.001 |

**We allowed for non-linear effects of log-10 viremia and age on the endpoints. To simplify interpretation of the results, ORs for two selected viremia and age contrasts from the models are presented.*

*^1^Since a number of interactions are present in the models, ORs and 95% CIs are shown for patients with age = 10, serotype = DENV-1, immune status = probable secondary infection, and illness day at enrolment = 3.*

*^2^Since a number of interactions are present in the models, the ORs and 95% CIs are shown for patients with log 10 viremia = 7.*

*CI: confidence interval; OR: odds ratio*

**Appendix 8. Results for the sensitivity analyses for inpatient/outpatient status at enrolment**

**Table S6. Relationships between variables and each of the three endpoints – sensitivity analysis including inpatient/outpatient status at enrolment (complete case analysis)**

|  | **Severe dengue** |  | **Plasma leakage** |  |
| --- | --- | --- | --- | --- |
| **Factor** | **OR (95% CI)** | ***P*** | **OR (95% CI)** | ***P*** |
| Log 10 viremia (copies/ml)^1^* |  | 0.001 |  | <0.001 |
| - 7 versus 6 | 1.15 (0.85 - 1.55) |  | 1.25 (1.01 - 1.56) |  |
| - 8 versus 7 | 1.07 (0.84 - 1.35) |  | 1.15 (0.98 - 1.34) |  |
| All interactions of log 10 viremia | - | 0.119 | - | 0.157 |
| Nonlinear effect of log 10 viremia | - | 0.010 | - | 0.519 |
| Age (years)^2^* |  | <0.001 |  | <0.001 |
| - 10 versus 5 | 1.08 (0.70 - 1.67) |  | 0.68 (0.53 - 0.88) |  |
| - 15 versus 10 | 0.64 (0.48 - 0.86) |  | 0.74 (0.65 - 0.84) |  |
| Non-linear effect of age | - | 0.076 | - | 0.573 |
| Serotype^2^ |  | 0.022 |  | 0.005 |
| - DENV-1 | 1 |  | 1 |  |
| - DENV-2 | 1.90 (1.23 - 2.94) |  | 1.64 (1.19 - 2.27) |  |
| - DENV-3 | 0.42 (0.18 - 0.98) |  | 0.79 (0.50 - 1.26) |  |
| - DENV-4 | 1.25 (0.73 - 2.13) |  | 1.06 (0.72 - 1.56) |  |
| Immune status^2^ |  | <0.001 |  | <0.001 |
| - Probable primary infection | 1 |  | 1 |  |
| - Probable secondary infection | 7.96 (3.06 - 20.70) |  | 2.47 (1.64 - 3.73) |  |
| - Indeterminate immune status | 1.43 (0.44 - 4.69) |  | 1.74 (0.90 - 3.33) |  |
| Illness day at enrolment (per one day increase)^2^ | 1.78 (1.42 - 2.22) | <0.001 | 1.81 (1.50 - 2.19) | <0.001 |
| Patient’s status at enrolment^2^ |  | 0.673 |  | <0.001 |
| - Outpatient | 1 |  | 1 |  |
| - Inpatient | 0.80 (0.51 - 1.26) |  | 2.26 (1.66 - 3.06) |  |

**We allowed for non-linear effects of log-10 viremia and age on the endpoints. To simplify interpretation of the results, ORs for two selected viremia and age contrasts from the models are presented.*

*^1^Since a number of interactions are present in the models, ORs and 95% CIs are shown for patients with age = 10, serotype = DENV-1, immune status = probable secondary infection, and illness day at enrolment = 3.*

*^2^Since a number of interactions are present in the models, the ORs and 95% CIs are shown for patients with log 10 viremia = 7.*

*CI: confidence interval; OR: odds ratio*

**Table S7. Relationships between variables and each of the three endpoints – sensitivity analysis including inpatient/outpatient status at enrolment (imputed data analysis)**

|  | **Severe dengue** |  | **Plasma leakage** |  |
| --- | --- | --- | --- | --- |
| **Factor** | **OR (95% CI)** | ***P*** | **OR (95% CI)** | ***P*** |
| Log 10 viremia (copies/ml)^1^* |  | 0.002 |  | <0.001 |
| - 7 versus 6 | 1.25 (0.96 - 1.63) |  | 1.29 (1.11 - 1.50) |  |
| - 8 versus 7 | 1.07 (0.84 - 1.35) |  | 1.26 (1.09 - 1.46) |  |
| All interactions of log 10 viremia | - | 0.589 | - | 0.982 |
| Nonlinear effect of log 10 viremia | - | 0.170 | - | 0.928 |
| Age (years)^2^* |  | <0.001 |  | 0.001 |
| - 10 versus 5 | 1.21 (0.80 - 1.84) |  | 0.86 (0.70 - 1.06) |  |
| - 15 versus 10 | 0.65 (0.49 - 0.88) |  | 0.84 (0.75 - 0.94) |  |
| Non-linear effect of age | - | 0.027 | - | 0.797 |
| Serotype^2^ |  | 0.097 |  | 0.024 |
| - DENV-1 | 1 |  | 1 |  |
| - DENV-2 | 1.67 (1.08 - 2.57) |  | 1.58 (1.18 - 2.13) |  |
| - DENV-3 | 0.42 (0.19 - 0.95) |  | 0.89 (0.55 - 1.43) |  |
| - DENV-4 | 1.12 (0.67 - 1.88) |  | 1.01 (0.73 - 1.39) |  |
| Immune status^2^ |  | <0.001 |  | <0.001 |
| - Probable primary infection | 1 |  | 1 |  |
| - Probable secondary infection | 8.14 (3.35 - 19.82) |  | 2.27 (1.57 - 3.27) |  |
| Illness day at enrolment (per one day increase)^2^ | 1.69 (1.37 - 2.09) | <0.001 | 1.09 (0.93 - 1.29) | 0.667 |
| Patient’s status at enrolment^2^ |  | 0.329 |  | <0.001 |
| - Outpatient | 1 |  | 1 |  |
| - Inpatient | 1.02 (0.66 - 1.59) |  | 2.65 (2.00 - 3.52) |  |

**We allowed for non-linear effects of log-10 viremia and age on the endpoints. To simplify interpretation of the results, ORs for two selected viremia and age contrasts from the models are presented.*

*^1^Since a number of interactions are present in the models, ORs and 95% CIs are shown for patients with age = 10, serotype = DENV-1, immune status = probable secondary infection, and illness day at enrolment = 3.*

*^2^Since a number of interactions are present in the models, the ORs and 95% CIs are shown for patients with log 10 viremia = 7.*

*CI: confidence interval; OR: odds ratio*

**Appendix 9. STROBE Statement - Checklist of items**

**Higher plasma viremia in the febrile phase is associated with adverse dengue outcomes irrespective of infecting serotype or host immune status: an analysis of 5642 cases**

|  | **Item No** | **Recommendation** | **Comments or page(s) reported** |
| --- | --- | --- | --- |
| **Title and abstract** | 1 | (*a*) Indicate the study’s design with a commonly used term in the title or the abstract | 1 |
|  |  | (*b*) Provide in the abstract an informative and balanced summary of what was done and what was found | 2 |
| **Introduction** | | |  |
| Background/rationale | 2 | Explain the scientific background and rationale for the investigation being reported | 3-4 |
| Objectives | 3 | State specific objectives, including any prespecified hypotheses | 4 |
| **Methods** | | |  |
| Study design | 4 | Present key elements of study design early in the paper | 5 |
| Setting | 5 | Describe the setting, locations, and relevant dates, including periods of recruitment, exposure, follow-up, and data collection | 5, Appendix 1 |
| Participants | 6 | (*a*) Give the eligibility criteria, and the sources and methods of selection of participants. Describe methods of follow-up | 5, Appendix 1 |
|  |  | (*b*) For matched studies, give matching criteria and number of exposed and unexposed | Not applicable |
| Variables | 7 | Clearly define all outcomes, exposures, predictors, potential confounders, and effect modifiers. Give diagnostic criteria, if applicable | 5-7, Appendices 2-3 |
| Data sources/ measurement | 8* | For each variable of interest, give sources of data and details of methods of assessment (measurement). Describe comparability of assessment methods if there is more than one group | 5-7, Appendices 2-3 |
| Bias | 9 | Describe any efforts to address potential sources of bias | 5-8 |
| Study size | 10 | Explain how the study size was arrived at | 5, 8 |
| Quantitative variables | 11 | Explain how quantitative variables were handled in the analyses. If applicable, describe which groupings were chosen and why | 6-8 |
| Statistical methods | 12 | (*a*) Describe all statistical methods, including those used to control for confounding | 7-8, Appendix 4 |
|  |  | (*b*) Describe any methods used to examine subgroups and interactions | 7-8 |
|  |  | (*c*) Explain how missing data were addressed | 7-8, Appendix 4 |
|  |  | (*d*) If applicable, explain how loss to follow-up was addressed | Not applicable |
|  |  | (*e*) Describe any sensitivity analyses | 8 |
| **Results** | | |  |
| Participants | 13* | (a) Report numbers of individuals at each stage of study—eg numbers potentially eligible, examined for eligibility, confirmed eligible, included in the study, completing follow-up, and analysed | 8, Figure 1 |
|  |  | (b) Give reasons for non-participation at each stage | 9, Figure 1 |
|  |  | (c) Consider use of a flow diagram | Figure 1 |
| Descriptive data | 14* | (a) Give characteristics of study participants (eg demographic, clinical, social) and information on exposures and potential confounders | 8-10, Table 1, Appendix 5 |
|  |  | (b) Indicate number of participants with missing data for each variable of interest | 8-10, Table 1 |
|  |  | (c) Summarise follow-up time (eg, average and total amount) | Not applicable |
| Outcome data | 15* | Report numbers of outcome events or summary measures over time | 9-10, Table 1 |
| Main results | 16 | (*a*) Give unadjusted estimates and, if applicable, confounder-adjusted estimates and their precision (eg, 95% confidence interval). Make clear which confounders were adjusted for and why they were included | 10-11, Table 2 |
|  |  | (*b*) Report category boundaries when continuous variables were categorized | Not applicable |
|  |  | (*c*) If relevant, consider translating estimates of relative risk into absolute risk for a meaningful time period | Not applicable |
| Other analyses | 17 | Report other analyses done—eg analyses of subgroups and interactions, and sensitivity analyses | 11-12, Appendices 6-8 |
| **Discussion** | | |  |
| Key results | 18 | Summarise key results with reference to study objectives | 12 |
| Limitations | 19 | Discuss limitations of the study, taking into account sources of potential bias or imprecision. Discuss both direction and magnitude of any potential bias | 13-16 |
| Interpretation | 20 | Give a cautious overall interpretation of results considering objectives, limitations, multiplicity of analyses, results from similar studies, and other relevant evidence | 13-16 |
| Generalisability | 21 | Discuss the generalisability (external validity) of the study results | 16 |
| **Other information** | | |  |
| Funding | 22 | Give the source of funding and the role of the funders for the present study and, if applicable, for the original study on which the present article is based | 16 |

*Give information separately for exposed and unexposed groups.

**Note:** An Explanation and Elaboration article discusses each checklist item and gives methodological background and published examples of transparent reporting. The STROBE checklist is best used in conjunction with this article (freely available on the Web sites of PLoS Medicine at http://www.plosmedicine.org/, Annals of Internal Medicine at http://www.annals.org/, and Epidemiology at http://www.epidem.com/). Information on the STROBE Initiative is available at http://www.strobe-statement.org.
